# Impact of diabetes mellitus on patients affected by oral lichen planus: a retrospective study

**DOI:** 10.3389/froh.2025.1569212

**Published:** 2025-03-31

**Authors:** Gianluca Tenore, Ahmed Mohsen, Andrea Ricciotti, Giordano Piombarolo, Gian Marco Podda, Cira Rosaria Tiziana Di Gioia, Umberto Romeo

**Affiliations:** ^1^ Department of Oral and Maxillofacial Sciences (SOMF), Sapienza University of Rome, Rome, Italy; ^2^ Department of Radiological, Oncological and Pathological Sciences, Sapienza University of Rome, Rome, Italy

**Keywords:** diabetes mellitus, dysplasia, oral manifestation, malignant transformation, oral cancer, oral lichen planus

## Abstract

**Introduction:**

The association between diabetes mellitus (DM) and oral lichen planus (OLP) has been widely reported. However, most of the studies focused on epidemiological aspects and shared inflammatory pathways, with few exploring the consequences of this association on the clinical course of OLP. The study aims to retrospectively observe the impact of DM on the clinical presentation and management strategy of OLP.

**Methods:**

A total of 97 OLP patients were retrieved from the Department database. The patients were distributed into two groups: OLP patients with DM “test group” (*n* = 47) and OLP patients without DM “control group” (*n* = 50). The descriptive and statistical analyses were performed on the variables related to the clinical presentation of OLP, the management of OLP, and the general and demographic information.

**Results:**

Regarding primary outcomes related to the clinical presentation variables, DM patients were symptomatic and more susceptible to present atrophic lesions at the first visit, compared to those without DM with a statistical significance (*p* = 0.0017 and *p* = 0.0016 respectively). Buccal mucosa was generally the most affected site in both groups and was notably higher in patients with DM (*p* = 0.0286). Regarding the management variables, DM patients were subjected to a higher number of follow-ups per year (*p* = 0.0420), a higher number of prescribed general treatments per year (*p* = 0.0006), and a higher number of prescribed non-cortisone-based treatments per year (*p* = 0.0001). In regard to the secondary outcomes related to the general and demographic variables, a statistically significant difference was observed with concomitant diseases, where patients with DM were more susceptible to concomitant diseases (*p* = 0.0321), particularly cardiopathy (*p* = 0.0422), arterial hypertension (*p* = 0.0418), dyslipidemia (*p* = 0.0411), and coagulopathy (*p* = 0.0411).

**Discussion:**

DM patients were highly presented with symptomatic OLP and showed a difference in the management strategy where more follow-ups and treatment prescriptions were needed. It seems that the clinician should consider DM as an essential co-factor that may influence the management procedures of OLP. Considering interdisciplinary management and involving endocrinologists may add significant value to the OLP management process.

## 1 Introduction

Oral lichen planus (OLP) is a common immune-mediated inflammatory disorder affecting the oral mucosa. Its prevalence ranges from 0.5% to 2% worldwide, primarily in middle-aged females. The pathogenesis of OLP is driven by immunological dysregulation involving CD4+ T helper and CD8+ T cytotoxic cells (1). Clinically, it manifests with exacerbation and remission phases, showing white reticular lesions with Wickham's striae accompanied or not by atrophic, erosive, bullous, ulcerative and/or plaque-type lesions. The lesions are frequently presented bilaterally in a symmetrical distribution in the oral cavity. OLP usually causes significant discomfort and may deeply affect the quality of life of the patients (2).

The histopathological features are the presence of a band-like lymphocytic infiltration in the lamina propria confined to the epithelium-lamina propria interface, hydropic degeneration of the basal cell layer, lymphocytic exocytosis, and the absence of epithelial dysplasia or verrucous epithelial architectural change. The definitive diagnosis of OLP should be based on the presence of both histopathological and clinical criteria (3, 4). In case of partial fulfilment of these criteria, the lesions should be considered as oral lichenoid lesions (3, 5, 6).

Clinically, OLP can be manifested in six different subtypes that can be categorized into two groups; the non-erosive/atrophic forms, which are usually asymptomatic and include the reticular form (the most common form), papular form, and plaque-like form. The second group comprises the atrophic-erosive forms, that are commonly symptomatic and associated with pain, burning, and/or soreness. This group includes bullous, atrophic, erythematous, erosive, and/or ulcerative lesions (7, 8). The asymptomatic presentation usually does not require pharmacological treatment, while symptomatic patients are usually managed with corticosteroid therapies such as triamcinolone acetonide, fluocinonide, and clobetasol propionate, as recommended by the European Academy of Dermatology and Venereology (EADV) (1). Despite ongoing research into new treatment options, such as innovative molecules or photo-biomodulation (1, 9), topical corticosteroids represent the first line of treatment in the management of symptomatic OLP after removing any potential irritants due to their efficacy and minimal adverse effects (10, 11).

Diabetes mellitus (DM) is a prevalent chronic metabolic disorder, affecting over 529 million individuals worldwide. There are two broad pathogenic DM categories: type 1 DM (T1DM) and type 2 DM (T2DM). However, this rigid classification cannot completely describe some diabetic patients due to the involvement of other genetic, immunological or neuroendocrinological pathways in the pathogenesis of DM. T1DM is caused by an absolute lack of insulin due to an immune-mediated destruction of at least 90% of pancreatic beta cells. The exact mechanism of its pathogenesis is still not completely understood (12). T2DM is the most common form of diabetes. It accounts for 90% of cases and reaches 24.4% of the global population aged 75–79 years (13). T2DM is characterized by insulin resistance and chronic low-grade inflammation (14).

In the literature, the role of inflammation in the pathophysiology of T1DM and T2DM has been frequently reported (12). This observation has led to an increase in research interest in targeting inflammation treatments in the prevention and management of DM (15). For the inflammatory role in T1DM, the synergic action of interferon-gamma (IFN-γ) and the innate inflammatory cytokines; tumor necrosis factor-alpha (TNF-α) and interleukin 1-beta (IL-1β), were suggested to play a role in the inflammation of pancreatic beta cells and consequent induction of specific gene-guided apoptosis of beta cells (12). In T2DM, some immunological changes were observed in adipose tissue, liver, pancreatic islets, the vascular system and circulating leukocytes. These immunological changes include alteration in the levels of specific cytokines and chemokines (e.g., interleukins, TNF-α, and adiponectin) and the number and activation state of various leukocyte populations which eventually result in increased apoptosis and tissue fibrosis. These observations together with some clinical trials with positive results using salicylates and IL-1 antagonists suggest that inflammation plays a role in the pathogenesis of DM and specifically T2DM (14, 15).

Some studies observed a significant elevation of the interleukin-8 (IL-8) serum levels in T2DM patients compared to healthy individuals (16). Generally, IL-8 plays an important role in activating neutrophils, chemotactic factors, T-cells, and basophils after injury, trauma, and/or inflammation. In OLP, following an increase in the local and systemic release of IL-1 and TNF-α, the keratinocytes, macrophages, T-cells, endothelial cells, and fibroblasts release significant amounts of IL-8, which eventually results in infiltration of T-cells, including cytotoxic T-cells, at the site of OLP lesions (16). These findings suggest that T2DM patients, who are characterized by higher serum levels of IL-8, may exhibit exacerbated manifestations of OLP due to this shared pathophysiological mechanism.

The association between OLP and DM was first reported by Grinspan in 1963 (17). Subsequently, several studies regularly reported this association. In a meta-analysis conducted by Mozaffari et al., it was observed that the prevalence of OLP in diabetic patients was twice as high as in non-diabetic individuals (18). This association has been frequently attributed to the above-mentioned role of IL-8, where patients with both DM and OLP showed high serum levels of IL-8 compared to patients who had neither both conditions nor one of the two (16). In addition, some studies demonstrated that OLP patients may exhibit altered glycemic profiles and considered OLP as a possible risk factor for the development of DM, suggesting a bidirectional relationship between the two conditions (19).

Despite several epidemiological and biochemical studies on diabetic patients affected by OLP, most of the studies were focused on the risk and prevalence of DM in OLP patients and to the best of our knowledge, few studies investigated the direct impact of DM on the clinical course and management strategies of OLP (19). Given the shared inflammatory basis by both diseases and the potential impact of hyperglycemia on tissue healing and immune response (20), it may be needed to study the correlation between OLP and DM and understand the impact of DM on the clinical presentation and management of OLP. The study aims to observe retrospectively the impact of DM on the clinical presentation and the management strategy of OLP by analyzing variables related to the clinical presentation, the management of OLP, and general and demographic information.

## 2 Material and methods

A hospital-based case-control retrospective study was conducted on patients of the oral surgery and MoMax units (Oral and Maxillofacial Medicine) of the Department of Oral and Maxillofacial Sciences, Sapienza University of Rome. The study was approved by the institutional review board of the department (Prot. N.2216, 09 December 2024). All the study procedures were performed following the ethical standards of the institutional and/or national research committee and with the 1964 Helsinki Declaration and its later amendments or comparable ethical standards. All the patients in the study signed an informed consent for participation in the research.

### 2.1 Data collection

The department's database and medical records were reviewed from 2016 to 2024. The inclusion criteria were patients with (1) a confirmed clinical and histological diagnosis of OLP according to the proposed diagnostic criteria of the American Academy of Oral and Maxillofacial Pathology (AAOMP), (2) a diagnosis of DM including type 1 and type 2, (3) a follow-up period of at least 6 months to ensure sufficient data for analysis, and (4) age ≥18 years (3). The exclusion criteria were (1) patients not fulfilling the inclusion criteria, (2) patients with other oral potentially malignant disorders (OPMDs), (3) patients with only clinical diagnosis of OLP, (4) patients with incomplete medical records and missing data, and/or (5) patients under supervision of another medical structure with previous clinical and histological diagnosis of OLP and the first biopsy in our department revealed the presence of oral squamous cell carcinoma (OSCC).

A total of 47 patients (18 males and 29 females) with an average age of 64.68 years old were found and fulfilled the study's inclusion criteria, 46 patients had T2DM and only one patient had T1DM. These patients were recruited into the test group (TG). A total of 50 OLP patients (16 males and 34 females) without the diagnosis of DM were selected with the same time search window and these patients were considered the control group (CG).

The collected data for both groups were: age (calculated at the first visit for clinical evaluation), gender, tobacco status, alcohol status, history of autoimmune disease, solid or hematological tumor history and any other concomitant diseases. For TG patients, the pharmacological treatment types of DM were collected.

Regarding the clinical features of OLP, the clinical presentation at the first visit, the site of the lesions and the total number of affected sites were retrieved from the patient's medical records. For the clinical presentation at the first visit, the patients were categorized into; patients with only white lesions, patients with any atrophic lesions without erosive lesions, patients with any erosive lesions with or without other clinical features “white and atrophic lesions”, and patients with bullous lesions with or without other clinical features “white, atrophic, and erosive lesions”. In addition, patients were further categorized into symptomatic and asymptomatic based on the presence or absence of symptoms at the first visit. For the site of the lesions, the oral cavity was defined as follows: buccal mucosa, gingiva, borders of the tongue, ventral surface of the tongue, dorsal surface of the tongue floor of the mouth, and hard and soft palate.

Regarding the management of OLP, the number of follow-ups per year, the total number of biopsies per year, the presence or absence of prescribed cortisone-based treatments (topical and/or systemic), the number of cortisone-based prescriptions received per year, and the number of non-cortisone-based prescriptions received per year were recorded for the patients of both groups.

In the case of observing dysplastic changes in the histological exams, the highest documented grade of dysplasia (low, moderate, and high) was registered. In patients with malignant transformation, the histological grading (G1, G2, and G3) and the site of the developed OSCC were registered. The sites of the developed OSCC were registered according to the 11th edition of the International Classification of Diseases (ICD-11), version 2019/2021 (21).

The primary studied variables of the study were the disease severity-related clinical data; including clinical presentation forms of OLP at the first visit and their sites, presence or absence of symptoms, number of follow-ups, number of performed biopsies, number of prescribed drugs (cortisone and non-cortisone based), the highest documented grade of dysplasia, and malignant transformation. The secondary studied variables were the patient's general profile-related data, including age, gender, smoking status, drinking status, presence or absence of other concomitant diseases, autoimmune diseases, and solid or hematological tumor history.

### 2.2 Statistical analysis

The patients' retrieved data were registered in an Excel sheet created using Office 365 (Microsoft Corporation, Redmond, WA, USA). The statistical analysis was performed using SPSS statistical processing software (Statistical Package for Social Science, Armonk, NY, USA) for Windows, release 25.0. The considered variables were divided into three categories: variables related to general and demographic information, variables related to the clinical presentation of OLP, and variables related to the management strategy of OLP. The continuous variables (age, number of OLP-affected sites, the total number of biopsies, number of biopsies per year, the number of received cortisone-based prescriptions per year, and the number of received non-cortisone-based prescriptions per year) were evaluated using the Shapiro test to assess their normality. To evaluate the impact of DM on OLP, DM patients (TG) and patients without DM (CG) were compared using the chi-square test for variables calculated on a nominal or ordinal scale (Fisher's exact test), *t*-test for independent samples, and Mann–Whitney test for metric variables. The odds ratio (OR) with a 95% confidence interval (95% CI) was calculated for all the considered variables in the study. The results were considered significant when the *p*-value was <0.05.

## 3 Results

### 3.1 Primary outcomes: clinical presentation features

A total of 50 patients (51.55%) had symptomatic lesions at the first visit. Among them, the most common clinical picture was atrophic lesions (*n* = 34) followed by erosive lesions (*n* = 15). Bullous lesions were found in only one patient. The most common affected sites were buccal mucosa (*n* = 68), gingiva (*n* = 32), and borders of the tongue (*n* = 24).

Comparing both groups, atrophic lesions (erythematous) at the first visit were significantly more frequent in patients with DM compared to those without DM (*p* = 0.0016). In addition, DM patients had an OR of 3.2 (95% CI: 1.2907–7.9334, *p* = 0.0006) for developing atrophic vs. white lesions. Symptomatic lesions at first visit were more present in patients with DM when compared to the patients without DM with a statistical significance of *p* = 0.0017 with OR of 3.793 (95% CI: 1.6334–8.8061). Buccal mucosa was generally the most affected site and was significantly higher in patients with DM (*p* = 0.0286). In contrast, the gingiva was highly affected in the patients without DM when compared to diabetic patients (*p* = 0.0564) (Table 1; Supplementary Table S1).

**Table 1:** An overview and comparison between test group “with diabetes mellitus (DM)” and control group “without DM” according to the clinical presentation features.

Variables	Total (*n* = 97)	Test group “with DM” (*n* = 47)	Control group “without DM” (*n* = 50)	*p*-value
Clinical presentation at first visit: *n* (%)				
White (reticular)	47 (48.45)	15 (31.91)	32 (64)	**0** **.** **0022**
Atrophic (erythematous)	34 (35.05)	24 (51.06)	10 (20)	**0**.**0016**
Erosive	15 (15.46)	7 (14.89)	8 (16)	1.0000
Bollous	1 (1.03)	1 (2.13)	0	0.4845
Symptomatic	50 (51.55)	32 (68.09)	18 (36)	**0**.**0017**
Asymptomatic	47 (48.45)	15 (31.91)	32 (64)	
Site of lesions: average (SD)	1.57 (0.88)	1.68 (0.75)	1.46 (0.97)	0.1260
Site distribution of lesions: *n* (%)				
Buccal mucosa	68 (70.10)	38 (80.85)	30 (60)	**0**.**0286**
Borders of the tongue	24 (24.74)	14 (29.79)	10 (20)	0.3474
Ventral surface of the tongue	12 (12.37)	6 (12.77)	6 (12)	1.0000
Dorsal surface of the tongue	3 (3.09)	2 (4.26)	1 (2)	0.6095
Floor of the mouth	5 (5.15)	3 (6.38)	2 (4)	0.6712
Hard and soft palate	8 (8.25)	5 (10.64)	3 (6)	0.4780
Gingiva	32 (32.99)	11 (23.40)	21 (42)	0.0564

*p*-values in bold are statistically significant.

### 3.2 Primary outcomes: management overview

For all the included patients, the average number of follow-ups was approximately 4 follow-ups per year. A total of 61 patients (62.89%) received topical treatments, 12 patients (12.37%) received systemic treatments, and 24 patients were not subjected to any treatments. The presence of dysplasia was observed in 37 patients (38.14%). Low-grade dysplasia was the most common type of dysplasia and was observed in 75.68% (*n* = 28) of all the OLP patients who showed dysplasia, 17 patients in TG and 11 patients in CG. Eleven patients (11.34%) showed malignant transformation, 6 patients in TG and 5 patients in CG. The tongue was the most affected site (*n* = 7, 63.64%), 5 patients in TG, and 2 patients in CG.

Comparing both groups, the statistical analysis revealed that DM patients showed a significantly higher number of follow-ups per year (*p* = 0.0420), a higher number of prescribed general treatments per year (*p* = 0.0006), and a higher number of prescribed non-cortisone-based treatments per year (*p* = 0.0001). No statistical difference was found between the two groups regarding the other considered variables; including the highest grade of dysplasia, the presence or absence of malignant transformation, the histological grading, and the site of the developed OSCC (Table 2; Supplementary Table S2).

**Table 2:** An overview and comparison between test group “with diabetes mellitus (DM)” and control group “without DM” according to the management overview.

Variables	Total (*n* = 97)	Test group “with DM” (*n* = 47)	Control group “without DM” (*n* = 50)	*p*-value
Number of follow-ups per year: average (SD)	4.24 (1.64)	4.6 (1.7)	3.9 (1.5)	**0** **.** **0420**
Number of biopsies per year: average (SD)	0.80 (0.69)	0.950 (0.82)	0.7 (0.5)	0.2386
Type of prescribed treatment: *n* (%)				
Topical	61 (62.89)	27 (57.45)	34 (68)	
Systemic	12 (12.37)	4 (8.51)	8 (16)	
No treatment	24 (24.74)	16 (34.04)	8 (16)	
Total number of prescribed treatments per year “both cortisone-based and non-cortisone-based treatments”: average (SD)	1.67 (1.20)	2.15 (1.35)	1.2 (0.8)	**0**.**0006**
Only cortisone-based per year: average (SD)	0.82 (0.93)	0.91 (1.13)	0.7 (0.7)	0.9528
Only non-cortisone based per year: average (SD)	0.84 (0.70)	1.22 (0.72)	0.5 (0.5)	**0**.**0001**
Presence of dysplasia: *n* (%)				
Yes	37 (38.14)	21 (44.68)	16 (32)	0.2165
No	60 (61.86)	26 (55.32)	34 (68)	
Highest grade of dysplasia: *n* (%)				0.6620
Low	28 (75.68)	17 (80.95)	11 (68.75)	0.1705
Moderate	4 (10.81)	2 (9.52)	2 (12.5)	1.0000
High	5 (13.51)	2 (9.52)	3 (18.75)	1.0000
Malignant transformation: *n* (%)				
Yes	11 (11.34)	6 (12.77)	5 (10)	0.7549
No	86 (88.66)	41 (87.23)	45 (90)	
Histological grading: *n* (%)				
G1	4 (36.36)	2 (33.33)	2 (40)	1.0000
G2	6 (54.55)	4 (66.67)	2 (40)	0.4297
G3	1 (9.09)	0	1 (20)	1.0000
Site of oral squamous cell carcinoma (OSCC): *n* (%)				
Tongue (2B62)	7 (63.64)	5 (83.33)	2 (40)	0.6797
Gingiva (2B63)	1 (9.09)	1 (16.67)	0	1.0000
Buccal mucosa (2B66)	1 (9.09)	0	1 (20)	0.4493
Palate (2B65)	1 (9.09)	0	1 (20)	0.4444
Floor of the mouth (2B64)	1 (9.09)	0	1 (20)	1.0000

*p*-values in bold are statistically significant.

### 3.3 Secondary outcomes: general and demographic information

The statistical analysis revealed that patients with DM were more susceptible to being affected by concomitant diseases (*p* = 0.0321), with an odds ratio (OR) of 3.777 (95% CI: 1.1333–12.5876, *p* = 0.0305). Cardiopathy showed a significant association with an OR of 4.469 (95% CI: 1.0545–18.9382, *p* = 0.0422). Similarly, arterial hypertension was associated with an OR of 3.674 (95% CI: 1.0492–12.8652, *p* = 0.0418), dyslipidemia with an OR of 13 (95% CI: 1.1092–152.3577, *p* = 0.0411), and coagulopathy with an OR of 13 (95% CI: 1.1092–152.3577, *p* = 0.0411). No significant difference was found with other considered variables; including age (at the first visit for clinical evaluation), gender, tobacco status, alcohol status, history of autoimmune disease, and solid or hematological tumor history (Table 3; Supplementary Table S3). Regarding the pharmacological treatments in DM patients, the majority of the patients were in treatment with metformin (*n* = 25, 53.19%), and eight patients (17.02%) were under treatment with more than one drug (Table 4).

**Table 3:** An overview and comparison between the test group “with diabetes mellitus (DM)” and the control group “without DM” according to the general and demographic information.

Variables	Total (*n* = 97)	Test group “with DM” (*n* = 47)	Control group “without DM” (*n* = 50)	*p*-value
Age: average (SD)	64.53 (11.62)	64.68 (11.34)	64.38 (11.99)	0.8312
≤60	34 (35.05)	18 (38.30)	16 (32)	0.5315
>60	63 (64.95)	29 (61.70)	34 (68)	
Gender: *n* (%)				
Female	62 (63.92)	29 (61.70)	33 (66)	0.6782
Male	35 (36.08)	18 (38.30)	17 (34)	
Tobacco status: *n* (%)				
Non-smokers	49 (50.52)	21 (44.68)	28 (56)	0.5332
Ex-smokers	31 (31.96)	17 (36.17)	14 (28)	
Smokers	17 (17.53)	9 (19.15)	8 (16)	
Alcohol status: *n* (%)				
Drinkers	7 (7.22)	3 (6.38)	4 (8)	1.0000
Non-drinkers	90 (92.78)	44 (93.62)	46 (92)	
Autoimmune disease: *n* (%)				
Yes	18 (18.56)	9 (19.15)	9 (18)	1.0000
No	79 (81.44)	38 (80.85)	41 (82)	
Type: *n* (%)				
Hashimoto's thyroiditis	5 (5.15)	3 (6.38)	2 (4)	
Psoriasis	2 (2.06)	2 (4.26)	0	
Sjogren's syndrome	2 (2.06)	0	2 (4)	
Hyperthyroidism	1 (1.03)	0	1 (2)	
Psoriatic arthritis	2 (2.06)	1 (2.13)	1 (2)	
Raynaud syndrome	1 (1.03)	0	1 (2)	
Pemphigus	1 (1.03)	0	1 (2)	
Ankylosing spondylitis	1 (1.03)	1 (2.13)	0	
Rheumatoid arthritis	1 (1.03)	0	1 (2)	
Asma	1 (1.03)	1 (2.13)	0	
Allergy	1 (1.03)	0	1 (2)	
Other concomitant diseases: *n* (%)				
Yes	80 (82.47)	43 (91.49)	37 (74)	**0** **.** **0321**
No	17 (17.53)	4 (8.51)	13 (26)	
Type: *n* (%)				
Cardiopathy	18 (18.56)	11 (23.40)	8 (16)	
Coagulopathy	5 (5.15)	4 (8.51)	1 (2)	
Arterial hypertension	49 (50.52)	26 (55.32)	23 (46)	
Osteoporosis	14 (14.43)	7 (14.89)	7 (14)	
Thyroid disease	25 (25.77)	12 (25.53)	13 (26)	
Viral infections (HCV, HBV, HPV, HZV, …)	5 (5.15)	3 (6.38)	2 (4)	
Dyslipidemia	5 (5.15)	4 (8.51)	1 (2)	
Prostate diseases	2 (2.06)	0	2 (4)	
Renal diseases	1 (1.03)	0	1 (2)	
Solid or hematological tumor history: *n* (%)				
Yes	10 (10.31)	5 (10.64)	5 (10.00)	1.0000
No	87 (89.69)	42 (89.36)	45 (90.00)	
Type				
Oral cancer	2 (2.06)	2 (4.26)	0 (0.00)	0.2416
Gastrointestinal cancer	4 (4.12)	3 (6.38)	1 (2.00)	0.3610
Breast cancer	2 (2.06)	0 (0.00)	2 (4.00)	0.4959
Prostate cancer	1 (1.03)	0 (0.00)	1 (2.00)	1.0000
Melanoma	1 (1.03)	0 (0.00)	1 (2.00)	1.0000
Cutaneous lichen planus: *n* (%)				
Yes	10 (10.31)	4 (8.51)	6 (12.00)	0.7416
No	87 (89.69)	43 (91.49)	44 (88.00)	

*p*-values in bold are statistically significant.

**Table 4:** Pharmacological treatment of diabetic patients.

Pharmacological treatment for diabetes mellitus:	*n* (%)
Not specified^a^	8 (17.02)
Insuline	2 (4.26)
Metformin	25 (53.19)
Sulfaniluree	1 (2.13)
Glucagon-like peptide-1 (GLP-1) agonist	3 (6.38)
More than one drug	8 (17.02)

^a^ Data were not specified in the medical charts due to the lack of reporting by patients.

## 4 Discussion

This retrospective study showed a more severe clinical manifestation of OLP in diabetic patients, suggesting a strong association between DM and OLP. The atrophic-erosive and bullous forms were more common in DM patients and the studied variables for the evaluation of the management process indicated a more complex course for DM patients.

From the general and demographic point of view, diabetic patients are known to have a wide array of systemic complications, including hypertension, cardiovascular disease, dyslipidemia, and obesity (22). These reported conditions and complications may themselves represent concomitant diseases or serve as risk factors for the development of additional pathologies. It was confirmed from our study that diabetic patients exhibited a significantly higher susceptibility to concomitant diseases (*n* = 43, 91.49%).

It has been hypothesized that OLP might present at an earlier age in diabetic patients. However, no significant difference was observed in the study related to the average age at the first visit when comparing OLP patients with and without DM. This result is in line with a cross-sectional study conducted by Sun and his colleagues, showing no differences in the average age (23). Also, no statistical difference was observed regarding other general risk factors such as smoking and alcohol status, that were reported as a relevant risk factor for the development of OSCC (24).

Few studies analyzed the clinical presentation of OLP in diabetic patients, with conflicting results. It was reported that the manifestations were much more severe and the erosive-atrophic form was more common in diabetic patients (25). Contrasting results were reported by Sun et al., where they found that the predominant form of OLP in diabetic patients was the white-reticular form, however, the results of this study were based on collected data of 12 patients (23). In our study, the atrophic (erythematous) forms at the first visit were significantly more common in OLP patients with DM (*n* = 24, 51.06%) compared to OLP patients without DM (*n* = 10, 20%).

The more severe form of OLP in DM patients might be due to the altered immunological spectrum and the immune dysregulation caused by DM that is the base of the OLP pathogenesis, where several routes may trigger the induction of keratinocyte apoptosis by CD8+ T-cells in OLP, one of those is the increased secretion of TNF-α and IL-6. In fact, elevated levels of TNF-α were reported in DM patients and IL-6 was considered to mediate adverse metabolic effects and contribute to insulin resistance (14, 26, 27).

Another explanation for the severe form of OLP in DM patients is endothelial dysfunction and microangiopathy, which were widely reported in the literature (28). Diabetic microangiopathy leads to a reduced peripheral blood supply in oral mucosa and consequently, this may induce higher cellular stress (29, 30). Similarly, in regards to OLP, Jana et al. reported higher cellular stress in erosive forms when compared to the reticular forms, which may highlight a kind of correlation between the two diseases (31).

In addition, the severity of OLP may be related to blood glucose control. The severity of microangiopathy in diabetic patients was observed to be closely linked to the control level of diabetes (28). Similarly, the risk of periodontitis and the severity of inflammation were reported as a possible consequence of hyperglycemia in diabetic patients (32). Blood glucose level is an important indicator for the treatment outcomes of DM. Poor blood glucose control can lead to oxidative stress and inflammatory reactions, which can increase susceptibility to oral tissue infections and local irritants and eventually cause a variety of oral-related complications, including oral mucosa lesions (33, 34). In a study conducted on DM patients with OLP, it was found that the increase in glycated hemoglobin (HbA1c) level was associated with an increased risk of OLP (35). This possible bidirectional relationship between the severity of OLP and the severity of DM may help physicians in the management of both diseases (27). The analysis of the HbA1c levels and other clinical parameters of DM in OLP patients might be helpful in future studies to confirm this hypothesis and explore the relationship between the clinical presentation of OLP and DM control levels (35).

Regarding the role of the pharmacological treatments of DM, in the literature, some authors observed positive feedback with effective topical treatment of erosive OLP on the glycemic level in diabetic patients (27). Moreover, effective management by pharmacological treatments was observed for both conditions of OLP and metabolic dysfunctions associated with DM (35). While no clear evidence is present for the correlation between the method or type of DM treatment and the development of OLP. Sun et al. studied the prevalence of OLP in DM patients and they did not find any association between hypoglycemic drugs or insulin and the OLP prevalence (23).

Regarding the localization of the OLP lesions, Scardina and his colleagues studied the oral mucosa of diabetic patients using capillaroscopy. They found a difference in endothelial dysfunction between the buccal mucosa and gingival regions (25). This may explain the more common presentation of OLP lesions on the buccal mucosa in diabetic patients. Similarly, it was found a greater tendency of OLP to manifest in the buccal mucosa in diabetic patients, where buccal mucosa and vestibular sulcus were involved in 83.3% of the patients (23).

The analysis of the obtained results of the study highlighted that the management of OLP in diabetic patients was considerably more complex than in non-diabetic patients. The average number of follow-up visits per year was significantly higher in diabetic patients (*p* = 0.0420). Similarly, the average number of biopsies performed per year, even if not statistically significant, was slightly higher in diabetic patients. These results may confirm the more severe clinical picture of OLP in diabetic patients during the follow-up process. This complexity may be also attributed to a combination of several factors, including more severe general clinical features, multiple comorbidities, and the need for extensive medication management (36, 37).

In addition, it was found in the study that diabetic patients required nearly double the number of total prescribed therapies (both cortisone and non-cortisone-based treatments). These data may confirm the higher severity of OLP in diabetic patients in the follow-up process. Interestingly, the analysis of the type of prescribed medications revealed a statistically significant difference with only the non-cortisone-based treatments, while the cortisone-based treatments were higher but without significance. This data may highlight that during the follow-up process, the diabetic patients showed more symptomatic conditions, which pushed the physicians to prescribe more non-cortisone-based treatments compared to non-diabetic patients.

Glucocorticoids exacerbate hyperglycemia through several mechanisms such as hepatic glucose production, protein catabolism, impaired insulin secretion, and glucose uptake of the peripheral tissues (38). This might lead clinicians to hesitate in prescribing corticosteroid therapies (both local or systemic) to diabetic patients, and sometimes the patients may not follow the prescribed therapies due to concerns about maintaining optimal glycemic levels.

The literature extensively documented an increased risk of cancer in diabetic patients, with studies reporting from 20% up to a 39% higher overall cancer risk (39, 40). The highest hazard ratios (HR) were observed for liver and pancreas cancers, while moderately increased risks were observed for oral, colon, gallbladder, reproductive (female), kidney, and brain cancers (40). Another research suggested an elevated risk of malignant transformation and oral cancer in individuals with diabetes (33, 41). The study outcomes did not reveal statistically significant differences regarding the malignant transformation between the diabetic and non-diabetic groups. A total of 6 diabetic patients showed a malignant transformation and 5 non-diabetic patients in the CG with no significant differences regarding the anatomical sites or histological grading of OSCC. However, the histological findings, regarding the presence or absence of dysplasia, revealed some considerable differences between the two groups of the study, even if not statistically significant, where the presence of dysplasia was observed in 21 diabetic patients (44.68%) and 16 non-diabetic patients (32%).

Some limitations and considerations were observed with this experience that need to be acknowledged; firstly, some clinical and laboratory parameters of DM were not available in the department medical records; such as glycemic levels and severity and clinical indicators of diabetes such as albuminuria, lipids and lipoproteins, ketonuria and arterial blood gas analysis (pH analysis, and ketonic bodies), due to the retrospective nature of the study. These parameters may give a more comprehensive overview of the possible associations between the severity of diabetes and the severity of OLP clinical manifestations. Secondly, only one patient of T1DM fulfilling the inclusion criteria of the study was found and included in the study, therefore, evaluating the different impact of both types of DM on OLP was not possible. Thirdly, the pharmacological treatment analysis did not reveal any correlation with other considered variables. This might be due to the small number of patients in each drug group. It also should be acknowledged that eight DM patients did not specify the pharmacological treatment. Fourthly, the initial intention was to include two cohorts of equal size, however, due to the limited availability of OLP patients with DM in the department database, it could not recruit 50 participants for the TG, while 50 patients with OLP were successfully enrolled. Fifthly, a potential bias should be considered since the data analysis was not conducted blindly. Finally, some patients with malignant transformation and clinical presentation of OLP were excluded due to not being followed up for at least 6 months after the final diagnosis of OLP. Therefore, it would be necessary to highlight these issues for a better interpretation of the study results and to consider them in future studies.

## 5 Conclusions

Based on the study results, DM patients were highly presented with symptomatic OLP and showed a difference in management strategy, with more follow-ups and treatment prescriptions needed. It seems that the clinician should consider DM as an essential co-factor that may influence the management procedures of OLP. Considering interdisciplinary management and involving endocrinologists may add significant value to the OLP management process. Further studies are needed to consider other variables, including the different types of DM and laboratory parameters, that may give a more comprehensive overview of the impact of DM on OLP patients.

## Data Availability

The raw data supporting the conclusions of this article will be made available by the authors, without undue reservation.
