# Supplementary material for: Impact of diabetes mellitus on patients affected by oral lichen planus: a retrospective study
Source: Front Oral Health. 2025 Mar 31;6:1569212. doi: 10.3389/froh.2025.1569212 (PMC11994681; doi:10.3389/froh.2025.1569212)
Supplement: Supplementary file 1 [file Table1.docx]

**Table S1.** Odd ratios (ORs) and their 95% confidence intervals (CIs) for the clinical presentation variables.

| **Variables** | **OR (95% CI)** | **p-value** |
| --- | --- | --- |
| **Clinical presentation at first visit:** |  |  |
| Atrophic (Erythematous) vs. white (Reticular) | 3.2000 (1.2907 - 7.9334) | **0.0006** |
| Erosive vs. white (Reticular) | 1.0000 (0.3062 - 3.2657) | 0.3024 |
| Bollous vs. white (Reticular) | 3.9796 (0.1553 - 101.9540) | 0.1573 |
| Symptomatic vs. asymptomatic | 3.7926 (1.6334 - 8.8061) | **0.0019** |
