# Supplementary material for: Impact of diabetes mellitus on patients affected by oral lichen planus: a retrospective study
Source: Front Oral Health. 2025 Mar 31;6:1569212. doi: 10.3389/froh.2025.1569212 (PMC11994681; doi:10.3389/froh.2025.1569212)
Supplement: Supplementary file 2 [file Table2.docx]

**Table S2.** Odd ratios (ORs) and their 95% confidence intervals (CIs) the management overview variables.

| **Variables** | **OR** (95% CI) | **p-value** |
| --- | --- | --- |
| **Presence of dysplasia:** |  |  |
| Yes vs. no | 1.7163 (0.7507 to 3.9240) | 0.2004 |
| **Highest grade of dysplasia:** |  |  |
| Low vs. no dysplasia | 2.0210 (0.8100 - 5.0424) | 0.1315 |
| Moderate vs. no dysplasia | 1.3077 (0.1725 - 9.9114) | 0.7952 |
| High vs. no dysplasia | 0.8718 (0.1356 - 5.6039) | 0.8851 |
| **Malignant transformation:** |  |  |
| Yes vs. no | 1.3171 (0.3736 - 4.6433) | 0.6684 |
| **Histological grading:** |  |  |
| G1 vs. no malignant transformation | 1.0976 (0.1478 - 8.1519) | 0.9275 |
| G2 vs. no malignant transformation | 2.1951 (0.3817 - 12.6240) | 0.3784 |
| G3 vs. no malignant transformation | 0.3655 (0.0145 - 9.2221) | 0.5411 |
| **Site of oral squamous cell carcinoma (OSCC):** |  |  |
| Tongue (2B62) vs. no malignant transformation in tongue | 2.0000 (0.3418 - 11.7033) | 0.4419 |
| Gingiva (2B63) vs. no malignant transformation in gingiva | 2.4146 (0.0922 - 63.2566) | 0.5967 |
| Buccal mucosa (2B66) ) vs. no malignant transformation in buccal mucosa | 0.2683 (0.0102 - 7.0285) | 0.4297 |
| Palate (2B65) vs. no malignant transformation in palate | 0.2683 (0.0102 - 7.0285) | 0.4297 |
| Floor of the mouth (2B64) vs. no malignant transformation in floor of the mouth | 0.2683 (0.0102 - 7.0285) | 0.4297 |
