# Supplementary material for: Impact of diabetes mellitus on patients affected by oral lichen planus: a retrospective study
Source: Front Oral Health. 2025 Mar 31;6:1569212. doi: 10.3389/froh.2025.1569212 (PMC11994681; doi:10.3389/froh.2025.1569212)
Supplement: Supplementary file 3 [file Table3.docx]

**Table S3.** Odd ratios (ORs) and their 95% confidence intervals (CIs) for the general and demographic variables.

| **Variables** | **OR** (95% CI) | **p-value** |
| --- | --- | --- |
| **Age:** |  |  |
| >60 vs. ≤60 | 0.7582 (0.3286 - 1.7493) | 0.5163 |
| **Gender:** |  |  |
| Female vs. Male | 0.8300 (0.3620 - 1.9027) | 0.6597 |
| **Tobacco status:** |  |  |
| Smokers vs. non-smokers | 1.5000 (0.4954 - 4.5414) | 0.4731 |
| Ex-smokers vs. non-smokers | 1.6190 (0.6544 - 4.0055) | 0.2971 |
| Smokers vs. ex-smokers | 0.9265 (0.2829 - 3.0343) | 0.8996 |
| **Alcohol status:** |  |  |
| Drinkers vs. non-alcohol drinkers | 0.7841 (0.1659 - 3.7053) | 0.7589 |
| **Autoimmune disease:** |  |  |
| Yes vs. no | 1.0789 (0.3875 - 3.0040) | 0.8844 |
| Type**:** |  |  |
| Hashimoto's thyroiditis vs. no | 1.6184 (0.2563 -10.2196) | 0.6086 |
| Psoriasis vs. no | 5.3896 (0.2507 - 115.8623) | 0.2819 |
| Sjogren's syndrome vs. no | 0.2156 (0.0100 - 4.6345) | 0.2156 |
| Hyperthyroidism vs. no | 0.3593 (0.0142 - 9.0884) | 0.5346 |
| Psoriatic arthritis vs. no | 1.0789 (0.0652 - 17.8635) | 0.9577 |
| Raynaud syndrome vs. no | 0.3593 (0.0142 -  9.0884) | 0.5346 |
| Pemphigus vs. no | 0.3593 (0.0142 -  9.0884) | 0.5346 |
| Ankylosing spondylitis vs. no | 3.2338 (0.1278 - 81.7955) | 0.4764 |
| Rheumatoid arthritis vs. no | 0.3593 (0.0142 - 9.0884) | 0.5346 |
| Asma vs. no | 3.2338 (0.1278 - 81.7955) | 0.4764 |
| Allergy vs. no | 0.3593 (0.0142 - 9.0884) | 0.5346 |
| **Other concomitant diseases:** |  |  |
| Yes vs. no | 3.7770 (1.1333 - 12.5876) | **0.0305** |
| Type**:** |  |  |
| Cardiopathy vs. no Concomitant diseases | 4.4688 (1.0545 - 18.9382) | **0.0422** |
| Coagulopathy vs. no Concomitant diseases | 13.0000 (1.1092 - 152.3577) | **0.0411** |
| Arterial hypertension vs. no Concomitant diseases | 3.6739 (1.0492 - 12.8652) | **0.0418** |
| Osteoporosis vs. no Concomitant diseases | 3.2500 (0.7009 - 15.0710) | 0.1321 |
| Thyroid disease vs. no Concomitant diseases | 3.0000 (0.7638 - 11.7830) | 0.1155 |
| Viral infections (HCV, HBV, HPV, HZV,…) vs. no Concomitant diseases | 4.8750 (0.5903 - 40.2592) | 0.1414 |
| Dyslipidemia vs. no Concomitant diseases | 13.0000 (1.1092 - 152.3577) | **0.0411** |
| Prostate diseases vs. no Concomitant diseases | 0.6000 (0.0240 - 14.9928) | 0.7557 |
| Renal diseases vs. no Concomitant diseases | 1.0000 (0.0343 - 29.1892) | 1.0000 |
| **Solid or hematological tumor history:** |  |  |
| Yes vs. no | 1.0714 (0.2894 - 3.9669) | 0.9177 |
| Type |  |  |
| Oral cancer vs. no | 5.3529 (0.2497 - 114.7456) | 0.2834 |
| Gastrointestinal cancer vs. no | 3.2143 (0.3216 - 32.1224) | 0.3201 |
| Breast cancer vs. no | 0.2141 (0.0100 - 4.5898) | 0.3243 |
| Prostate cancer vs. no | 0.3569 (0.0141 - 9.0021) | 0.5315 |
| Melanoma vs. no | 0.3569 (0.0141 - 9.0021) | 0.5315 |
| **Cutaneous lichen planus:** |  |  |
| Yes vs. no | 0.6822 (0.1798 - 2.5875) | 0.5739 |
